# Microstructure Optimization of Mg-Alloys by the ECAP Process Including Numerical Simulation, SPD Treatments, Characterization, and Hydrogen Sorption Properties

**DOI:** 10.3390/molecules24010089

**Published:** 2018-12-27

**Authors:** Nataliya Skryabina, Valery Aptukov, Petr Romanov, Daniel Fruchart, Patricia de Rango, Gregory Girard, Carlos Grandini, Hugo Sandim, Jacques Huot, Julien Lang, Rosario Cantelli, Fabrice Leardini

**Affiliations:** 1 Physics Department, Perm State University, Bukireva 15, Perm 614990, Russia; natskryabina@mail.ru; 2 Mathematics Department, Perm State University, Bukireva 15, Perm 614990, Russia; aptukov@psu.ru (V.A.); petr_rom@yahoo.com (P.R.); 3 Institut Néel, CNRS & UGA, BP 166-38042 Grenoble Cedex 9, France; patricia.derango@neel.cnrs.fr (P.d.R.); gregory.girard@mcphy.com (G.G.); 4 Lab. de Anelasticidade e Biomateriais, Universidade Estadual Paulista, 17.033-360 Bauru, SP, Brazil; beto_grandini@pq.cnpq.br; 5 Department of Materials Engineering, Lorena School of Engineering, EEL-USP, 12602-810 Lorena, Brazil; hsandim@demar.eel.usp.br; 6 Institut de Recherche sur l’Hydrogène, Université du Québec à Trois Rivières, 3351, boul. des Forges, C.P. 500, Trois-Rivières, QC G9A 5H7 Canada; jacques.huot@uqtr.ca; 7 Canadian Nuclear Laboratories, Material Sciences Branch, Chalk River, ON K0J-1J0, Canada; julien.lang@cnl.ca; 8 Dipartimento di Fisica, Sapienza Università di Roma, 00185 Roma, Italy; rosario.cantelli@roma1.infn.it; 9 Departamento de Física de Materiales, Universidad Autónoma de Madrid, Campus Cantoblanco, 28049 Madrid, Spain; fabrice.leardini@uam.es

**Keywords:** magnesium alloys, severe plastic deformation, ECAP process, numerical simulation, grain and crystallite size, texture, anelastic spectroscopy, hydrogen uptake and kinetics sorption

## Abstract

Both numerical simulation and hardness measurements were used to determine the mechanical and microstructural behavior of AZ31 bulk samples when submitted to the Equal Channel Angular Pressing (ECAP) technique. Billets of this representative of Mg-rich alloys were submitted to different numbers of passes for various ECAP modes (anisotropic A, isotropic B_C_). The strain distribution, the grain size refinement, and the micro-hardness were used as indicators to quantify the effectiveness of the different processing routes. Structural characterizations at different scales were achieved using Scanning Electron Microscopy (SEM), micro-analysis, metallography, Small Angle Neutron Scattering SANS, X-Ray Diffraction (XRD), and texture determination. The grain and crystallite size distribution and orientation as well as defect impacts were determined. Anelastic Spectroscopy (AS) on mechanically deformed samples have shown that the temperature of ECAP differentiate the fragile to ductile regime. MgH_2_ consolidated powders were checked for using AS to detect potential hydrogen motions and interaction with host metal atoms. After further optimization, the different mechanically-treated samples were submitted to hydrogenation/dehydrogenation (H/D) cycles, which shows that, for a few passes, the B_C_ mode is better than the A one, as supported by theoretical and experimental microstructure analyses. Accordingly, the hydrogen uptake and (H/D) reactions were correlated with the optimized microstructure peculiarities and interpreted in terms of Johnson-Avrami- Mehl-Kolmogorov (JAMK) and Jander models, successively.

## 1. Introduction

In recent years, evolution of the microstructure of metals and alloys submitted to Severe Plastic Deformation (SPD) processes was the subject of numerous studies [1,2,3,4,5,6]. This was stimulated by the opportunity using SPD to create unique mechanical properties for new and already known applications. Among the various SPD methods, Equal Channel Angular Pressing (ECAP) appears to be one of the most effective and affordable process. However, more recent and detailed information on all SPD techniques applied to M-H systems can be found in refs. [7,8] from the authors.

In ECAP deformed samples, ultra-fine grain (UFG) refinement can be realized up to less than 100 nm, depending on the nature of the processed metal materials, since the processing parameters can be changed for better understanding [9]. In this case, our interests focus on magnesium and its alloys with an hcp symmetry (less studied up to date) more effectively for the ultra-fine grain (UFG) and crystallite refinements expecting fast hydrogenation kinetics in forming MgH_2_ [10,11,12,13,14,15]. Early on, very wide developments were made for years by using Ball-Milling (BM) techniques and the huge numbers of papers report excellent results in forming MgH_2_ [16,17,18,19,20,21,22,23,24]. However, it must be underlined that the BM technique remains difficult to upscale because it requires too much energy, time, and man-power consumption. Therefore, the first aim of the present study is to better understand both experimental and numerical analyses regarding how a minimum number of ECAP passes could be necessary and sufficient to optimize a fine microstructure of Mg alloys. This can readily form MgH_2_.

ECAP was proposed for the first time by V.M. Segal who patented the method, noticing that under the conditions of channeling, the sample can accumulate large deformations without a noticeable change of size in all three directions [25,26]. In the early 1990s, R.Z. Valiev and collaborators extended the works using ECAP as well as other SPD techniques. The main principles of the ECAP method can be found in books and papers such as ref. [25,26,27,28,29]. Since ECAP allows multiple passing cycles, it results in the accumulation of shear deformations with sample structure refinement based on volume. E.g., successive cycles of ECAP can be carried out without changing the orientation of the sample relative to its initial position (A mode) or turning the sample by ±90° (В_А_, В_С_ modes) or even turning the sample by 180° (C mode) [28]. Depending on the nature of the material and its mechanical characteristics, in particular at hardening, ECAP can be performed in selected temperature ranges. Since it is important to preserve the integrity of the sample when applying a multi-cycle process, a proper temperature range should be chosen accordingly. If the ECAP deformation temperature is higher than the recrystallization temperature, weak effectiveness in grain refinement can be achieved [30,31]. Furthermore, depending on the crystal structure (bcc, fcc, hcp…) of the considered material, different types of slip band systems must be accounted for, which plays a role in the final mechanical characteristics gained after ECAP processing.

During the investigations and optimization of the ECAP modes to deliver ultra-fine structures of Mg, AZ31, and ZK60 alloys, it was found that the minimum grain size can be obtained at 200 °С for a B_C_ ECAP pass [32,33]. In this case, the deformation process was accompanied not only by the grain refinement, but also by increasing the level of internal stresses. Similar results were reported in ref. [32] where it was demonstrated that, for the formation of a fine-grained structure of the alloy Ma2-1 (which chemical composition is very close to that of AZ31), only two ECAP passes with orthogonal channeling are effective. The efficiency for a double ECAP process maximizes the grain refinement of a cubic material, which was reported in ref. [28].

Recently, AZ31 type alloys [33,34] were submitted to ECAP deformation and a critical analysis of the resulting mechanical properties was made versus the number of passes. However, in the research studies mentioned above, the SPD treatments were applied at 573 and 623 K, respectively. The temperatures are significantly higher than those mentioned in ref. [35], which leads to dynamical recrystallization, a regime not considered here.

Microstructure (SEM and Metallography), XRD (size of crystallites, stress level), and X-ray texture analyses have allowed parallel and consistent characterizations. Some of our preliminary results have been reported in refs [36,37,38]. Two Mg-rich alloys treated by ECAP (mode B_C_) apart from the range of fragile/ductile transformation were submitted to Anelastic Spectroscopy (AE) analysis versus the temperature for different vibration frequencies, which differentiates the response of the deformed materials. The MgH_2_ consolidated powder specimen were analyzed as well by using AE in order to check the hydrogen diffusion process. Then hydrogen absorption/desorption cycles were systematically realized on the different mechanically treated magnesium alloys in order to establish the best correlation between the deformation mode and microstructure refinement as well as the kinetics of hydrogen absorption/desorption and the final hydrogen uptake.

## 2. Results

### 2.1. Pre-Optimizing ECAP Processing from Numerical Analysis

**Figure 1:**
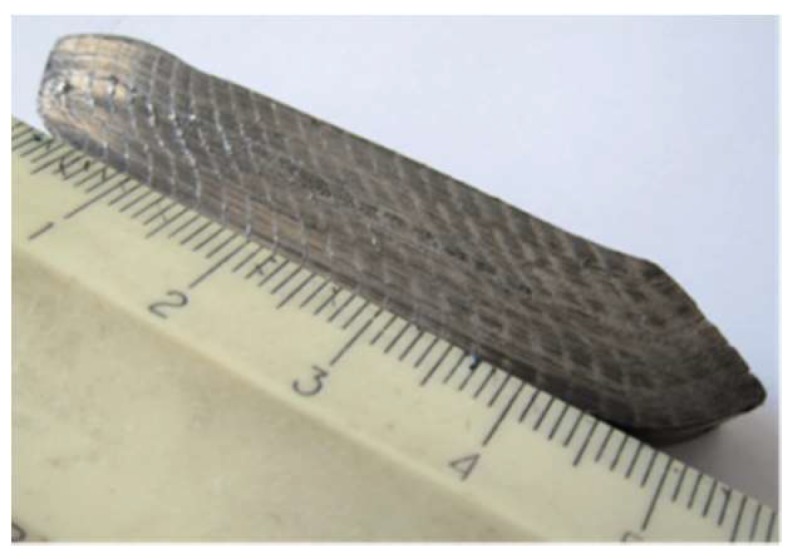
A picture of ECAP processed billet shows transformation of a reference square array drawn on the four 50 × 10 mm faces at the initial state.

**Figure 2:**
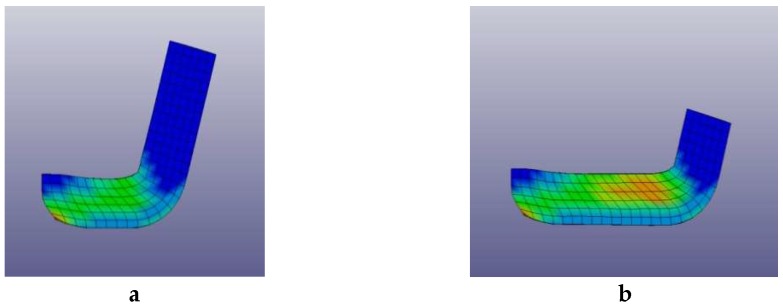
Simulated distribution of strain intensities: (**a**) at 1/3 pass, (**b**) at 3/4 pass of the 1st ECAP pass (relative scales: blue = minimum strain, green = intermediate strain, red = maximum strain).

In-samples strain fields established during the 1st ECAP pass were calculated as well as after the 2nd and 3rd cycles of deformation using a numerical simulation model detailed in Section 4: Materials and Methods. Figure 1 displays the state of strain fields after a first pass reference to the initial square lattice through ECAP die, which was retained for the numerical analyses shown in Figure 2a,b.

According to these pictures, the strain intensity distribution in the sample is inhomogeneous. There is an area of the billet close to the upper surface of the die channel, which reveals a high level of stresses. If the numerical analysis results are compared to experimental investigations of the microstructure of the AZ31 alloy, it becomes apparent that the size of grains of the billet after only one ECAP process is extremely non-uniform. 

**Figure 3:**
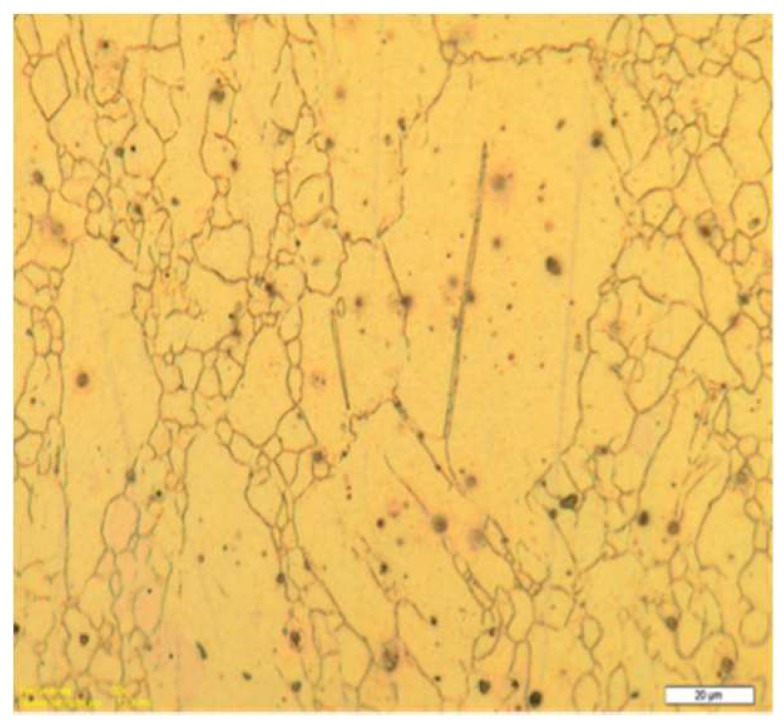
Microstructure of a pure Mg sample after 1 ECAP pass at RT. The white bar scale is for 20 µm.

Figure 3 shows a metallography picture of a pure Mg billet after one ECAP pass shows large grain sizes. The microstructure is quite similar to the one achieved with the AZ31 alloy. It suggests that the deformation of the sample was realized via two main mechanisms: 1st—a twinning process where large grains maintain their initial shape and 2nd—formation of new large-angle boundaries, i.e., formation of new grains due to the destruction of the larger ones near the existing boundaries.

**Figure 4:**
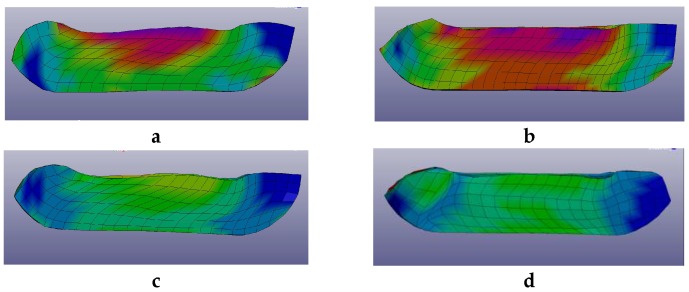
Strain intensity distribution after 2 ECAP passes done: (**a**)—along mode A, (**b**)—along mode B_C_, after 3 ECAP passes done: (**c**)—along mode A, (**d**)—along mode B_C_. The relative scale is between minimum strain = blue, intermediate strain = green and maximum strain = red without any physical meaning.

The presence of non-uniform strains within the billet is also shown by the micro-hardness measurements on the plate samples cut from different regions of the billet perpendicularly to the channeling ECAP axis. Measurements were made on the perimeter of the square sample close to the billet surface and at the center of the plate near the axis of the die channel. The average values of micro-hardness in the central zone of the billet were found a little larger than those at the edges of the cross section. This feature demonstrates the non-uniform nature of stress/strain distribution inside the billet. The calculations reveal that the distribution of stress after the 2nd and 3rd ECAP pass depends on the orientation of the billet relative to its initial position. This corresponds to the experimental modes А or В_С_. The results of numerical simulations are shown in Figure 4a–d.

**Figure 5:**
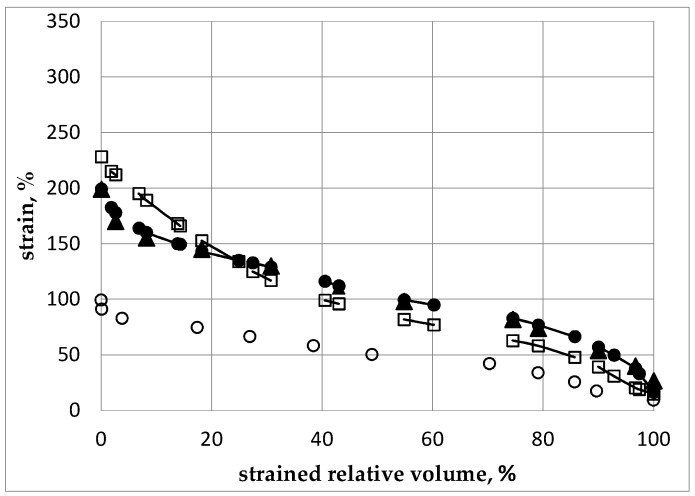
Strain level (%) versus the strained relative volume of sample (%) after different ECAP processes—open circles (O): after a 1st pass, open squares (**□**): after a 2nd pass, billet is not rotated reference to the 1st pass, black circles (**●**): after a 2nd pass, with a rotation of *φ* = 90° reference to the 1st pass, black triangles (▲): after a 2nd pass, with a rotation of *φ* = 180°.

**Figure 6:**
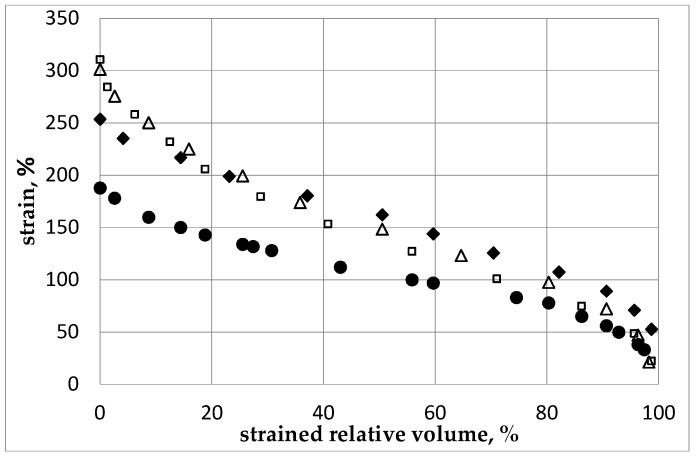
Strain level (%) versus the strained relative volume of sample (%) after the 3rd pass with different rotations relative to the 2nd pass. Black dots (**●**): optimal ECAP process (Figure 5) with rotation of *φ* = 90° relative to the 1st pass (В_С_ mode). Open squares (**□**): billet was not rotated relative to the 2nd pass. Black diamonds (♦): rotation is *φ* = 90° relative to the 2nd pass. Open triangles (∆) rotation is *φ* = 180° relative to the 2nd pass.

The strain distribution in the billet appears substantially non-uniform, i.e., different regions exhibit significantly different levels of residual strains. Thus, to compare the levels of accumulated strains in the material after different modes of ECAP, we have considered the strained relative fraction of the sample percentage in the total volume versus the strain intensity. For a given minimum, volume of sample corresponding to a strained relative fraction and strain intensity coordinate in Figure 5, it means that the corresponding strain intensity is at least higher than the strained relative fraction (%). The strain by volume distributions after subsequent passes is shown in Figure 6 for different ECAP modes for 1 pass and then for 2 passes after the billet was rotated along its axis by a given angle, *φ* being 0° (initial orientation), 90°, and 180°, successively.

The numerical analysis shows that the highest level of local strains in the billet can be achieved by ECAP processing, according to the А mode during the 2nd pass. However, according to Figure 5 and Figure 6, the corresponding volume fraction with the maximum achievable strains remains rather low. Moreover, the strain distribution appears highly non-uniform by volume. The best option, in terms of level and homogeneity of accumulated strains during a second pass, was achieved after rotating the billet by *φ* = 90°. The black dots (Figure 5 and Figure 6) correspond to the В_С_ mode. For practical interests, it is important that an average value of strain can be installed in more than half of the material volume and, accordingly, a low strain level concerns a limited volume only.

As shown in Figure 6, after the 3rd ECAP pass, the increase of strains reference to the second pass is not marked in the comparison with the gain obtained between the first and the second ECAP process. There is a substantially non-uniform strain distribution by volume of billet. Hence, from numerical simulation, it results that, regardless of the amount of operated ECAP cycles, a fully uniform distribution of the strain level cannot be achieved throughout the material volume. As a primary outcome and for practical interest, it is necessary to specify in what volume of the billet the average value of the strain level was achieved as well as what maximum strain level can be achieved versus the number of the ECAP process. In the present case, after the first pass, about 50% of the sample volume reached a value of strain intensity larger than 50% in which the maximum level is ~95–100%.

After the 2nd pass, about 50% of the sample has reached a value of strain intensity larger than 80%–110% depending on the φ angle of rotation applied to the sample. In such a case, the maximum of the strain level was found to be up to 200%–230% even though the concerned volume was insignificant. After the 3rd pass, about 50% of the sample has reached a value of strain intensity not less than 150%–170% (depending on the rotation angle) and the maximum level of strain has reached 250%–300%. In this case, the values are reported in terms of plastic strain percents accumulated in the material relative to the initial state where the plastic strain level is said to be equal to zero. However, it is worth noticing that the increment of strains decreases from the 1st to the 2nd and then to the 3rd cycle. Plotting the changes of strain distribution in the billet all along the successive passes i.e., the 1st to 2nd to 3rd ECAP processes lets it appear that, for all cycles with *φ* = 0° (А mode), the strains accumulate mainly in the area close to the upper surface of the channel, i.e., the microstructure refinement should occur mostly in this area. This feature can explain the lower efficiency in terms of total strained volume of the mode А when compared with the mode В_С_. As pointed out above, operating a single ECAP pressing at room temperature does not lead to maximizing the grain refinement as shown in Figure 3, which results in a very heterogeneous microstructure within the billet. To refine the grains further, the ECAP process must be repeated several times. However, further channeling the workpiece at room temperature becomes difficult since cracks are initiated within the billets [38,39]. Consequently, in order to evaluate the impact of an increasing number of ECAP cycles on the level of accumulated strains, heating of the billet must be operated, which allows successive ECAP cycles. Therefore, the operating temperature of at least 200 °C was selected. 

**Figure 7:**
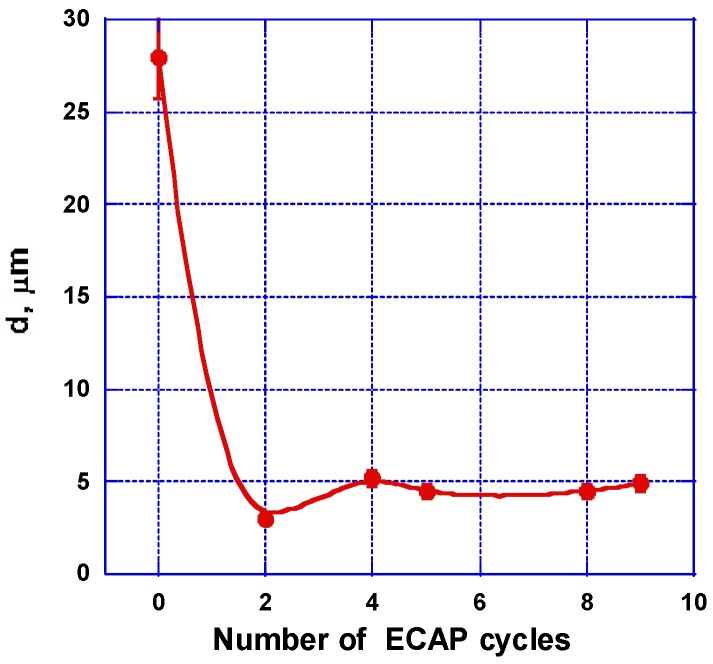
Impact of the number of ECAP passes on the grain size in a AZ31 billet as processed at 200 °C. It is worth it to note that, after 3 passes, a minimum grain size of 3 µm was achieved and then stabilized.

In fact, at 200 °C, the grain refinement results in deformation mechanisms [37,38,40,41] while it is not related to the recrystallization phenomena. In fact, the two mechanisms lead to significant differences in the final mechanical properties of magnesium-based materials. The studies demonstrate that, when increasing the number of deformation cycles, the grain size decreases up to its minimum value obtained after 2 В_С_ passes, which is shown in Figure 7. Increasing the number of cycles leaves the grain size practically unchanged, which is in agreement with the results of ref. [9].

**Figure 8:**
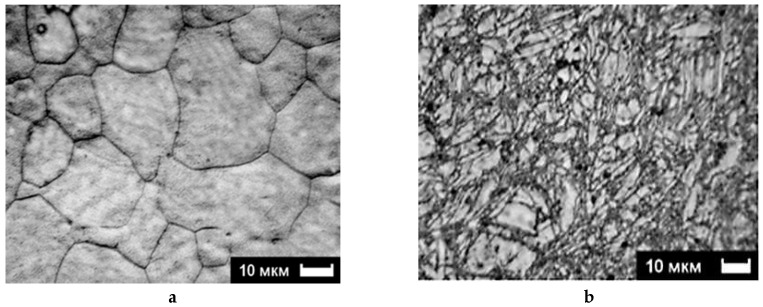
AZ31 microstructure: (**a**)—initial state, (**b**)—after 2 B_C_ passes at 200 °C.

**Figure 9:**
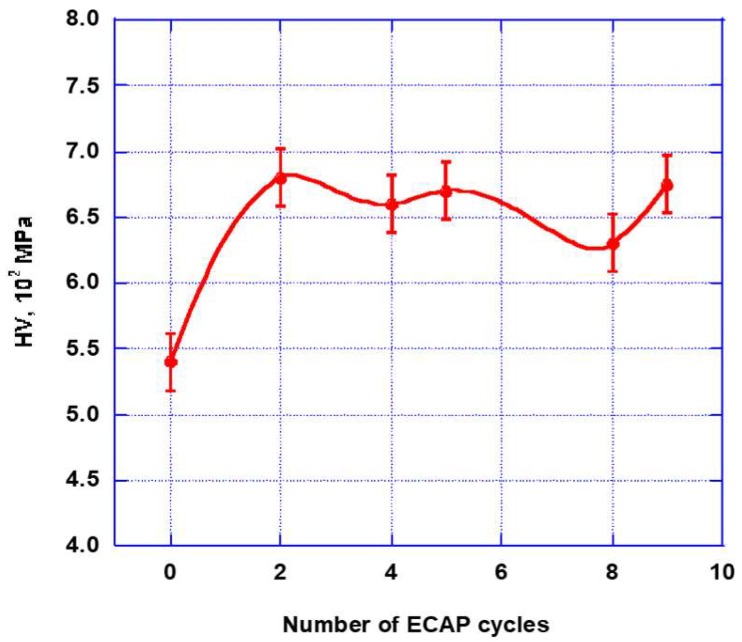
Microhardness of the AZ31 alloy vs the increasing number of ECAP passes at 200 °C.

In principle, microhardness should increases in parallel with the increasing number (decreasing size) of grains, according to Figure 8 and Figure 9. Actually, this suggests that the main mechanism driving the microhardness implementation is the increase of the grain boundary lengths while, at the applied temperature of 200 °C for ECAP deformation, the stress level contribution should be considered almost negligible. Specifically, at this point, a certain difference between the statements of numerical and experimental approaches may be underlined.

The microstructure analysis reveals typical features when resizing and shaping the grains. Figure 8a,b show SEM pictures of the original state of the alloy and after two ECAP cycles operated at 200 °C, respectively.

In the original state, the grains show equal axis shapes with classic triple joint features. After the ECAP deformation, a very strong grain refinement appears, which is not only accompanied by the formation of new high angle boundaries, but also by a large amount of deformation twins [9,36,42]. As shown in Figure 9, it is found that, after the 2nd ECAP pass, the microhardness increased by ~25%, i.e., showing a significant hardening of the material. After applying further cycling, one can notice fewer marked changes of the microhardness have occurred near an average value of ~6.6 × 10^2^ MPa.

**Figure 10:**
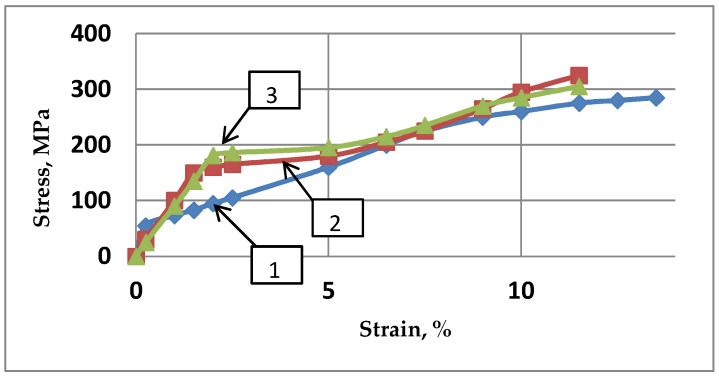
Dependence of the stress/strain response during ECAP compression of the AZ31 samples at RT: 1, as-cast (♦); 2, after 1st ECAP pass (■); 3, after 2nd ECAP pass (▲).

When studying the structure transformation of a real material, and conversely to a numerical simulation approach, a mechanism responsible for deformation of operating principles implies first dislocation slip systems typical for a given crystal structure. However, the stress relaxation operates at much higher temperatures. For the subsequent experiments, the temperature operating for ECAP was chosen lower but not too far from the recrystallization temperature of the processed alloy. Close to the threshold of the recrystallization temperature, the hardening level decreases due to relaxation of the internal stresses and the annihilation of dislocations. Effectively, it should be accounted that during energetic ECAP operation, the over-heating of the sample can be as large as 40–50 °C [38]. If one analyzes the data of microhardness and the strain level increase results from the application of numerical methods, it should be underlined that, for a direct comparison with the experimental parameters, some extrinsic characteristics are accounted for. Experimental compression of the magnesium alloy before and after ECAP at room temperature [35,43] reveals significant changes of the mechanical performances and, accordingly, a qualitative change in the stress-strain curve is shown in Figure 10.

After the 1st ECAP pass, the yield strength was increased by 2.7–2.8 times as well as the tensile strength with increases limited to 1.15–1.4 times only. However, after a 2nd pass, the tensile strength was no more increased and the average yield strength was increased by 13% while the elastic modulus was decreased by 8%–11%. 

**Figure 11:**
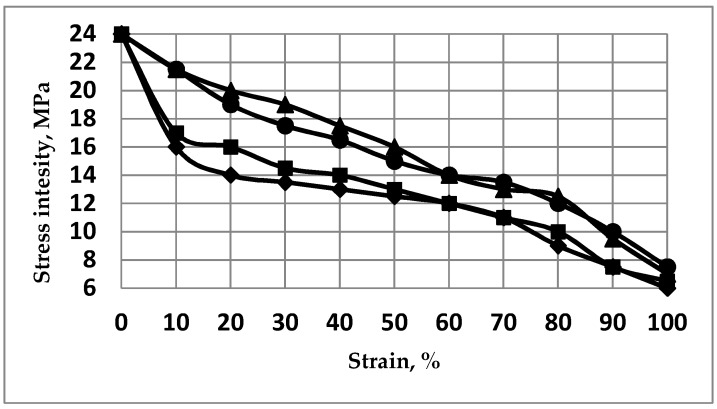
Strain level (%) versus the strained relative volume of the sample (%) versus the ECAP passes: diamonds (♦): 1st pass, triangles (▲): 2nd pass at *φ* = 90°, being the optimal combination, mode B_C_. Black squares (■): 2nd pass at *φ* = 180°, mode A, black circles (**●**): 3rd pass at *φ* = 90° relative to the 2nd pass at *φ* = 90°, mode B_C_.

Thus, the as-cast AZ31 alloy exhibiting strong strain hardness was transformed to a less hardened alloy after the ECAP operation with a well-defined yield line. For the numerical calculation analysis, only “cold” stress-strain diagrams were considered. All results illustrate the behavior of the stress-strain state for room temperature passes of the samples along the rigid matrix considering accumulated plastic strain and the self-balanced field of residual stresses. The distribution functions of the train level (%) within the strained relative volume versus the intensity of stresses as calculated after several passes are shown in Figure 11. The calculations show that, during the sequence from an optimized 2nd pass (operated at different *φ* angles) to the third, the level of residual stress, which is controlled by the level of achieved yield stress, did not increase.

### 2.2. Experimental Microstructure Analysis: Grain Refinement, Defects, and Texture

#### 2.2.1. SEM and Optical Microscopy

As shown in Figure 3, as done on pure Mg with the benefit of an initial larger grain size, the phenomenon is quite similar to what was achieved with the AZ31 alloy. The deformation of the sample after one pass (~A mode) at room temperature was realized via two main mechanisms. There is a twinning process within the large grains that have maintained their initial shape if well-oriented and parallel by forming new large-angle boundaries. There is a formation of new grains due to fragmentation of those of the larger grains’ setting near the already existing borders.

**Figure 12:**
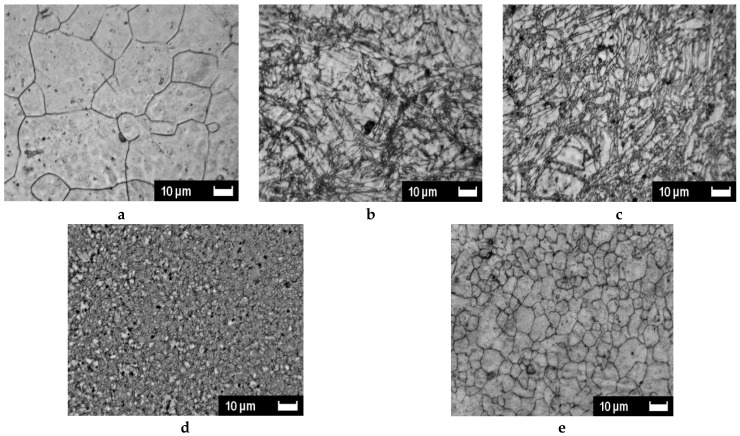
Microstructure versus temperature of AZ31 billets after 2 B_C_ ECAP passes: (**a**)—as received, (**b**)—150 °C, (**c**)—200 °C, (**d**)—250 °C, (**e**)—300 °C.

**Figure 13:**
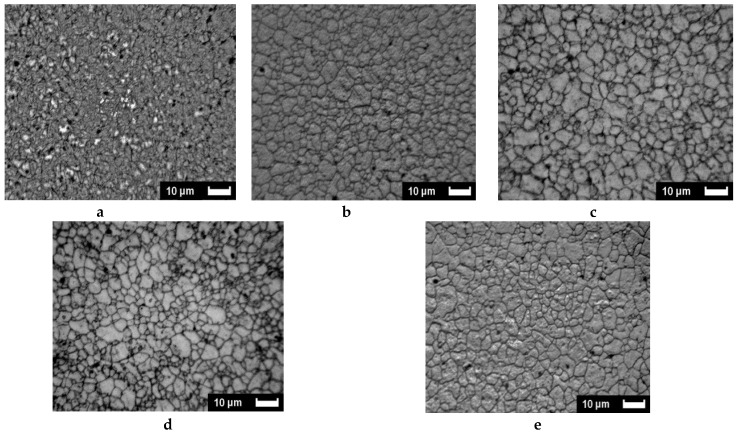
Microstructure versus the number of B_C_ ECAP passes of AZ31 billets after ECAP B_C_ passes at 250 °C: (**a**)—2 passes, (**b**)—4 passes, (**c**)—5 passes, (**d**)—8 passes, (**e**)—9 passes.

The SEM-analyzed microstructures of AZ31 materials treated in mode B_C_ in order to quantify the progression of the SPD treatments are presented versus temperature first (Figure 12) and then versus the number of applied passes (Figure 13). This analysis refers to the rather rapid and more homogeneous development of stresses in a sample, which was demonstrated by numerical analysis considering the B_C_ mode. Figure 12 shows the successive pictures recorded after two passes of B_C_ treatment at 150, 200, 250, and 300 °C successively compared to the initial state. It appears that the initial well shaped grains of 10–40 µm size are first roughly fractured (170 °C). Then the fractures are made denser (200 °C) and a very fine and homogeneous microstructure is achieved at 250 °C. Furthermore, at a higher temperature (300 °C), the SPD treatment induces a fast recrystallization process, which delivers a rather homogeneous distribution of well-formed grains of ~1 to 3 µm size.

**Figure 14:**
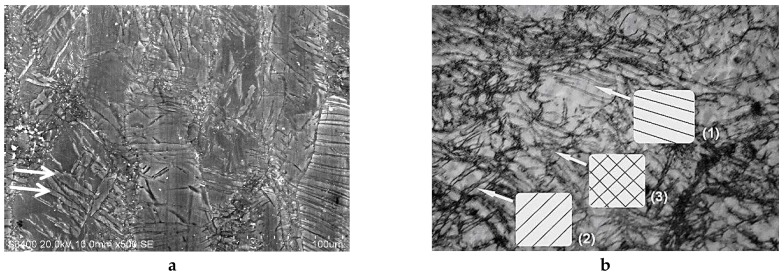
Microstructure and twinning: (**a**): ZK60 for 1 pass A mode at 150° C: various fields of twins affecting large and well oriented grains, (**b**): AZ31 for 2 passes B_C_ mode at 150 °C where zones can be seen as twin orientations along the 2 successive passes directions of (1) and (2) and (3) perpendicular bi-twinned grains.

In Figure 13, the number of B_C_ passes varied from 2 to 9 by applying the ECAP process at 250 °C where a fine size of homogeneously distributed grains was achieved after two passes. It is seen that, at this temperature, adding further passes leads that anticipate the fast recrystallization process, which was observed for two passes only but at higher temperature (300 °C). As already reported occurring for one pass (Figure 3) with Mg samples, the ECAP process allows us to develop two types of defects through the mechanism, which leads to the development of a fine grain structure, provided that the temperature remains low enough to avoid the fast recrystallization process. This was underlined above with Figure 3. On Figure 14, micrograph pictures reveal fields of twin boundaries created in large grains of ZK60 submitted 1 pass and AZ31 submitted to 2 ECAP passes mode B_C_ at 150 °C (Figure 14a,b, respectively). 

In Figure 14b, two fields (1) and (2) of primary twins can be distinguished as oriented in perpendicular directions. A third one (3) accommodates the two directions of stress fields. This should be a typical result for a large grain transformation. Mixed zones with twins “networks” and random cracks of reduced size (dislocation fields) can be seen as a general trend and are already shown in Figure 3 (Mg at RT, 1 pass) and in Figure 8 (AZ31, B_C_, 2 passes at 200 °C).

#### 2.2.2. XRD Analyses: Crystallite Size and Stresses Distribution

**Figure 15:**
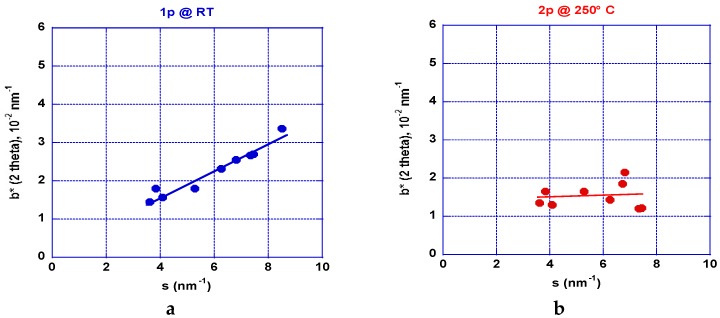
Williamson-Hall [44] plots allow for the study of the impact of temperature processing AZ31 billets submitted to A-type ECAP passes: (**a**)—marked strained material with a mean small size of crystallites of 300-400 nm for 1 pass at RT, (**b**)—2 passes at 250 K, which revealed almost no strain in spite of a marked anisotropic distribution and a minimum mean size of crystallites (<100 nm).

**Figure 16:**
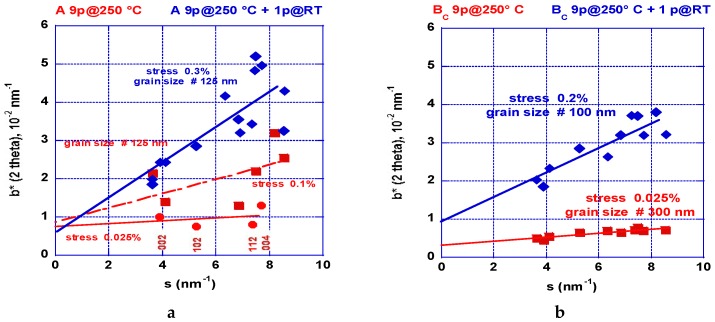
Williamson-Hall [44] plots allow for the determination of the crystallite size and the level of stresses leading modulation of the linewidth of XDR patterns versus the Bragg angle (scattering vector s) for ECAP-treated AZ31 billets at 250 °C: (**a**)—mode A - 9 passes, red: (dots and squares: see text), 9 passes + 1 pass at RT (blue), (**b**)—mode B_C_ - 9 passes (red), 9 passes + 1 pass at RT (blue).

The XRD analyses on the different AZ31 samples received after the ECAP processing were recorded on the square section of the bars (cut perpendicular to the channeling). The bars were cut using a wire saw and the surface was polished mechanically using progressively fine grit sand papers and then etched by using a specific electrolytic method and by applying the same process as used to prepare the surface for optical metallography analysis. The data were recorded in a reflexion mode using a D5000 (λ_Kα(Co)_ = 1791 Å) diffractometer (Brucker, Karlsruhe, Germany) equipped with a graphite (002) backscattering monochromator. After application of the Williamson-Hall formula, resulting analyses are shown in Figure 15a,b and Figure 16a,b [44].

Figure 15a shows the results received after one ECAP pass at room temperature. The deformation appears fairly homogeneous. Figure 15b shows the results received after two ECAP passes undertaken at 250 °C. The level of stresses looks to be weaker but it appears not fully homogeneous. Since we know from the numerical simulation analysis that more ECAP processes are mandatory, we have compared processing on mode A and B_C_ applied at 150, 200, 250, and 300 °C. The number of passes depends on the applied temperature since the material can behave too brittle. However, this number was progressively increased up to nine passes at 300 °C. Figure 16a shows the results gained e.g., after nine ECAP passes mode A at 250 °C and a 10th pass was realized at RT. After the first nine passes, the level of stresses is found quite anisotropic: the basal planes (002) and (004) reveal a small stress level. Conversely, the other planes show a marked inhomogeneous distribution and more stresses.

After application of the RT final ECAP pass, the level of stresses has increased, which is rather similar to that shown in Figure 15a but, in this case, the dispersion is marked. Figure 16b shows the results concerning mode B_C_ realized in the same conditions as the previous ones. At 250 °C, the level of stresses was found fairly homogeneous contrary to what was obtained after two passes (Figure 15b). The well-defined slope remains weak. After application of a final ECAP pass at RT, the level of stresses was found rather homogeneous even when taking a higher level, but less than for the sample processed in mode A.

The temperature of 250 °C appears quite critical because being close to the recrystallization temperature and the grains exhibit fine and homogeneous sizes, which is seen in Figure 12 and Figure 13. Under these conditions, the deformation more freely operates via the basal plane (e.g., Figure 12: red squares = in plane, red dots = out of plane) [42] and, consequently, the level of stresses remains weak. As observed, the mode B_C_ leads to a more homogeneous distribution of stresses than the mode A, which is in agreement with the numerical simulation analysis. In fact, the B_C_ mode operates more rapidly leading to a more homogeneous distribution of stresses and defects within the billet for intermediate (175 °C) and high temperature treatments (275 °C). However, final low temperature ECAP treatments e.g., at RT, lead to a (re)increase of the level of stresses. Effectively, as shown later, this “final” treatment was applied systematically to deliver brittle matter for hydrogen sorption measurements since the PCI experiment requires easy powdering materials.

#### 2.2.3. Texture Analysis

**Figure 17:**
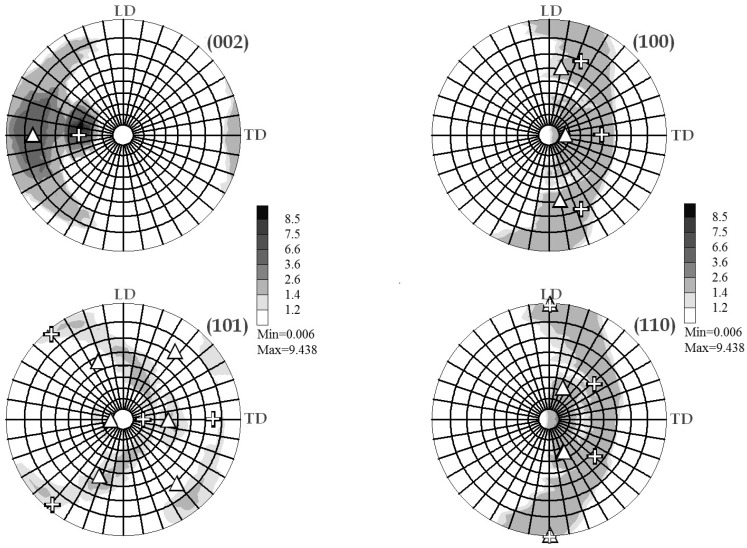
Stereographic projections of pole figures for the planes: (002), (100), (101), and (110).

As for the conventional XRD analysis, the texture investigations were conducted on the perpendicular cross section on the billet. Figure 17 shows the stereographic pole figure (ϕ_S_, ρ_S_) recorded from different plane reflections after one ECAP pass of the AZ31 sample.

The maximum pole is deviated at ρ_S_ ~40° from the axis perpendicular to the analyzed surface, which is shown in Figure 17a and corresponds to the (002) plane, according to the stereographic coordinate ρ_S_ = 90 − Φ_E_/2 where Φ_E_ = 105° (ECAP channeling angle). The other pictures of Figure 17b–d are addressed to the other crystal main planes (100), (101), and (110), respectively, which revealed only weak texture effects with a wide scattering distribution on the stereographic projection similarly to a Debye-Scherrer distribution.

**Figure 18:**
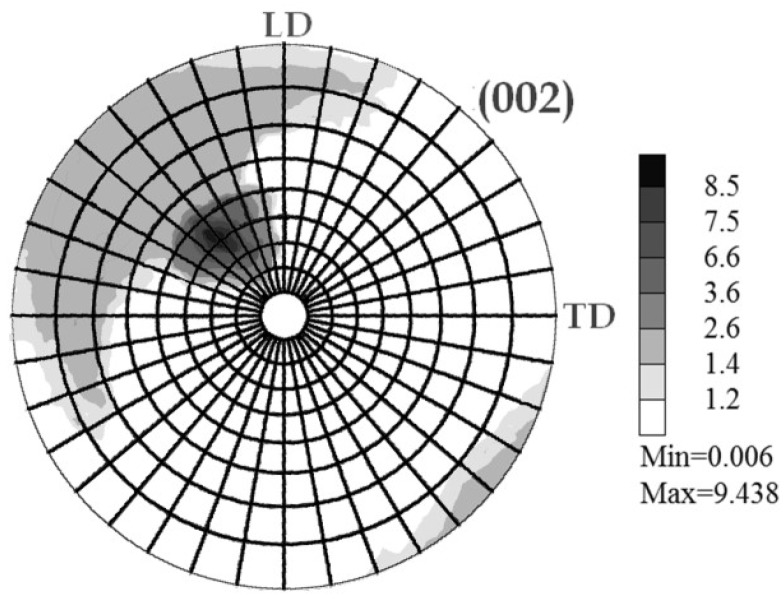
Pole figure of the (002) plane obtained after 2 passes mode B_C_.

Since the sample was rotated by 90° in between pass 1 as shown Figure 17a and pass 2 as shown as shown Figure 18, the resulting maximum pole figure for (002) was found rotated by ϕ_S_ = 45° reference to the initial one pass state (~mode A).

**Figure 19:**
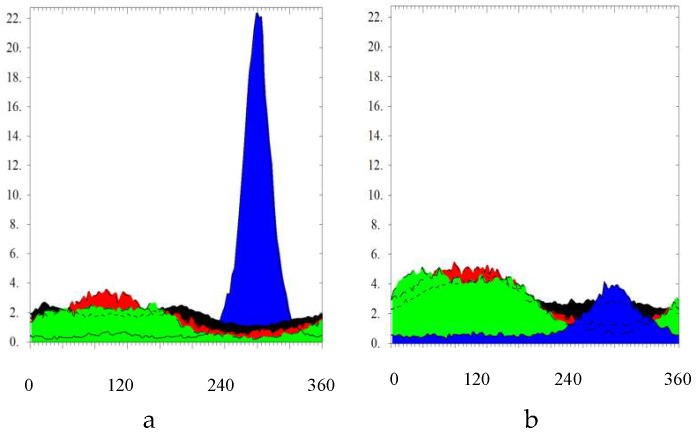
Texture integrated and normalized intensities of the four main crystallographic planes. (**a**)—after 1 pass (~A), (**b**)—after 9 passes B_C_. Colors are for: blue (002), red (100), black (101), and green (110) lines.

Figure 19a,b shows the integrated intensities from the pole figures of the four main reflection planes (002), (100), (101), and (110) after summation around ρ_S_ (i.e., the revolution symmetry axis or azimuthal angle) for 1 ECAP and 9 ECAP passes along B_C_ mode. The fully anisotropic intensity distribution observed for one pass (~A mode) shown in Figure 19a demonstrates the preferential of the grain (002)-axis along the channeling. All the other main directions appear homogeneously distributed. After a number of ECAP passes (≥3) in B_C_ mode, the texture of the material is made almost isotropic, as shown in Figure 19b leading to redistribution of the grain orientations but as well of the defects caused by the stresses accumulated, e.g., the twins at the boundaries.

#### 2.2.4. Small Angle Neutron Scattering (SANS)

**Figure 20:**
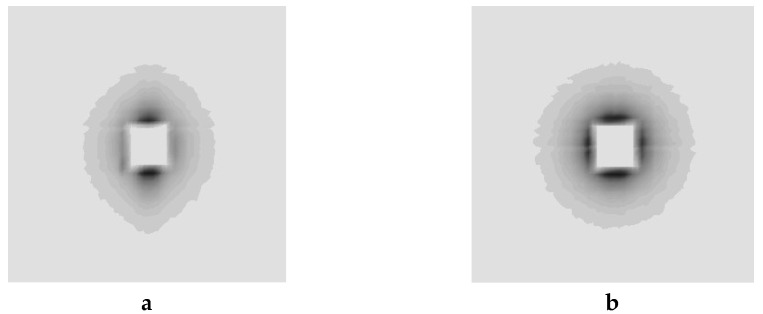
Small angle neutron scattering records (halo) of 1 pass AZ31 at RT for (**a**)—39 and (**b**)—8 m as distances sample to detector.

The experiments performed at Institute Laue-Langevin, Grenoble, France, using the D11 [45] instrument were made on 10 × 10 × 1 mm pieces cut as before in AZ31 billets after being submitted to 1 and 2 to 4 ECAP process in the B_C_ mode at room temperature, which shows oval shapes for the diffusing entities. The diffuse intensity versus the scattering vector Q extracted from patterns shown Figure 20, was expressed by using a Guinier type formula [46], accounting or not for anisotropic dispersion terms (GRASP and SASfit codes). In the range 0.1 to 0.4 nm, the scattering vector exhibits a Q-4 vector dependence using an exponent −3.65 for better fitting the data, for which most of the neutron dispersion occurs on non-smooth surfaces. Among several models allowing the interpretation of the diffusion data from the I(Q) dependence of small angle intensity vs the scattering vector Q. The best fit results are from ellipsoidal-type entities. One of the ellipsoid axes should range from 310 to 360 nm and the other lengths approximately of ~100 nm. Additionally, the results are better explained in terms of thin wall spheroids with a wall thickness approximately of 4–8 nm that can be caused by inhomogeneity (decoration of the grain boundaries either by dislocation fields or by Al-Zn precipitates or even both).

#### 2.2.5. Anelastic Spectroscopy (AS) on Alloys and MgH_2_ Powders

First, one has to recall that MgH_2_ does not exist as the bulk material but forms more or less fine decrepitated powders depending on the fabrication process. It is well known that the intrinsic diffusion coefficient of hydrogen in Mg is as low as K = 1.11 × 10^−8^ m^2^/s [47] at about 500 K. According to NMR [48] results with different MgH_2_ samples (un-milled and milled with various additives), the relaxation rate of the H motion in MgH_2_ appears too fast to be measured by AS by using a vibration frequency in the kHz range, where it can be detected. Attempts were made by using our MgH_2_ powders compressed in the form of 3.5 × 6 × 40 mm bars in a dye under up to 15 T. They were covered with a thin layer of silver paint and mounted on the nodal lines of the first flexural vibration mode to be investigated first in the temperature range from 300 to 500 K, as shown in ref. [49]. The plot of the elastic energy dissipation factor (Q^−1^) does not reveal any peak singularity and the relative variation of the dynamic Young modulus (ΔE/E) presents a monotonic increase when the temperature decreases. Then it was observed that Q^−1^ values remains almost temperature-independent until 650 K. Above that temperature, MgH_2_ decomposition is observed and produces an increase in Q^−1^ values as well as a change in the dynamic Young modulus, which is a phenomenon due to MgH_2_ to Mg phase transformation. This agrees with the NMR analyses applied to diffusion processes in MgH_2_ [47,48].

Two bulk pieces of pure commercial Mg were processed by AS after being submitted to 4 ECAP passes B_C_ mode at 175 °C and 225 °C, respectively, which is called Mg#2 and Mg#1. Two AZ31 samples were prepared in the same conditions, but, unfortunately, their surfaces revealed too much micro-cracked features to be correctly characterized by AS vibrations. The measurements made on Mg#2 and Mg#1 were realized when heating and cooling down between 300 and 475 K and successively applying vibration frequency of 1, 3, 10, 25, and 50 Hz, according to ref. [50]. The elastic dissipation factor was found to vary smoothly and increase (decrease) when the temperature increases (decreases) in the Q^−1^ range (0.01–0.02). The overall variations of the Young modulus were found to be similar, which shows a monotonic increase (decrease) when the temperature decreases (increases).

**Figure 21:**
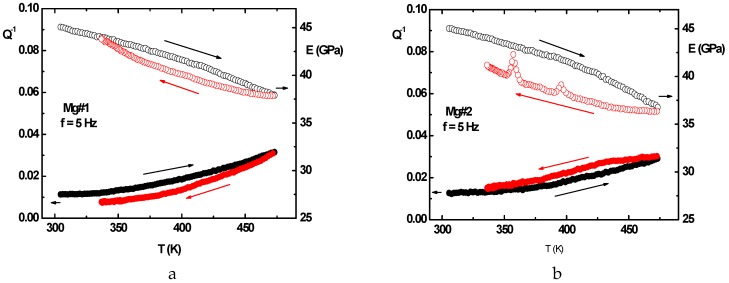
Anelastic spectroscopy traces recorded under 5 Hz excitation, of the elastic energy dissipation factor Q^−1^ (dots) and Young modulus E (open circles) versus temperature (heating = black, cooling down = red) shown e.g., for (**a**) (left) Mg#1 sample (225 °C, B_C_ ECAP treated) and (**b**) (right) for Mg#2 sample (175 °C, B_C_ ECAP treated).

However, the hysteretic difference between the heating and cooling down branches for Mg#2 (ECAP at 175 °C) is more than double of that for Mg#1 (ECAP at 225 °C). Again, for the Mg#1 sample, the cooling down branches of Q^−1^ takes place lower than the heating branch, which is contrary to what is observed for the Mg#2 sample. Interestingly, another difference in terms of the Young modulus behavior comes from two peaks appearing at 355 and 395 K on the cooling down branches of Mg#2, which does not exist with Mg#1, as shown Figure 21a,b.

**Figure 22:**
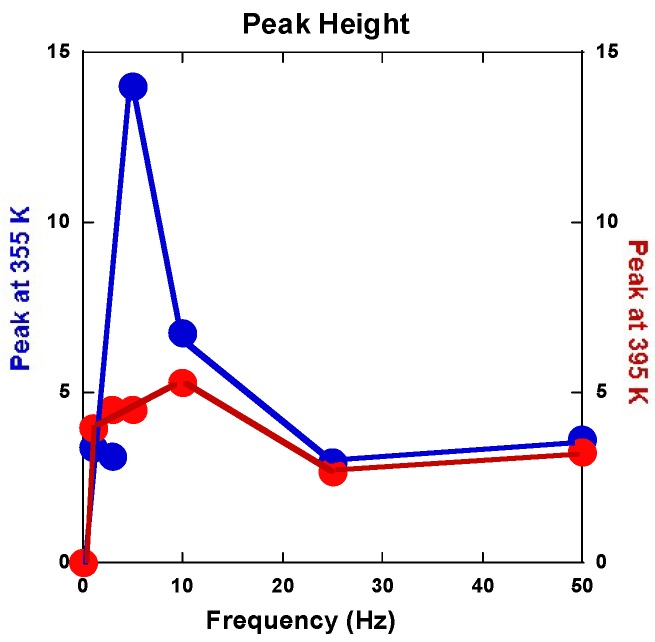
Relative height of the two peaks found on cooling down branch Mg#2 4 B_C_-175 °C. Lines are guides for the eyes (origin dots have no physical meaning).

The variations of intensity of these two anomalous peaks versus frequency are reported in Figure 22. Such a phenomenon on the Young modulus is somewhat surprising since, correspondingly, no anomaly appears on the elastic energy dissipation branches as shown ref. [51]. Furthermore, both the anomalous peak behaviors appear correlated versus the applied frequency.

However, the main peak at 355 K should correspond to what is reported in ref. [52] and considered as {10–12} twinning motion or (and) 2nd order pyramidal <c + a> slips but not motion in the hexagonal basal planes as <a> slips [53]. At the highest temperature, gliding grain boundaries in the material should be accounted for. Anyway, such a typical behavior must be studied more deeply when checking different sample types (Mg, AZ31, ZK60…) after being submitted to different ECAP processes and being realized at different temperatures.

### 2.3. Hydrogen Uptake and Kinetics

PCI traces were recorded on the differently ECAP processed AZ31 samples. Two modes of deformation were considered for the A anisotropic repetitive channeling and the “alternate orientation” B_C_ channeling said isotropic. A number of 1 to 9 passes was applied at various temperatures. In this case, we selected the 3 and 8 passes treated samples to compare the effectiveness of the deformation process in terms of hydrogen reactivity. In order to simplify again the present lecture, only two of the processing temperatures are considered for 175 °C down to T_F_ the fragile-to-ductile transformation of AZ31 and 275 °C, which is above T_F_. Since the ECAP-treated samples were made brittle but not especially des-aggregated, a short time treatment of the still bulk but fragile materials was realized using a SPEX miller with 5 w% in addition to MgH_2_, which led to the disposal of coarse powder to be placed in the PCI sample holder. The conditions applied for hydrogen sorption were 2 MPa H_2_-pressure and temperature of 350 °C for absorption and under 10 kPa for desorption. These values fulfill rather well the operating conditions we have applied on real MgH_2_-based hydrogen storage test-tank [22,23,24].

Before examining the successive ECAP activated samples, we like to recall that, according to the given conditions above, a fresh sample absorbs almost linearly ~0.5 w% hydrogen in 15 h and, after 24 h, exposed to H_2_ pressure it desorbs almost linearly ~0.6 w% hydrogen within 2 h (with a remains of ~0.25 w% hydrogen).

#### 2.3.1. Route A

**Figure 23:**
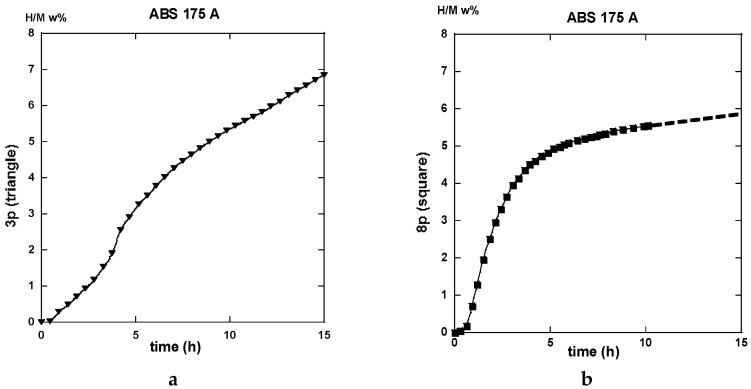
Hydrogen absorption rate (H/M w%) versus time for the samples treated in mode A at 175 °C (black): (**a**)—for 3 passes, (**b**)—for 8 passes.

**Figure 24:**
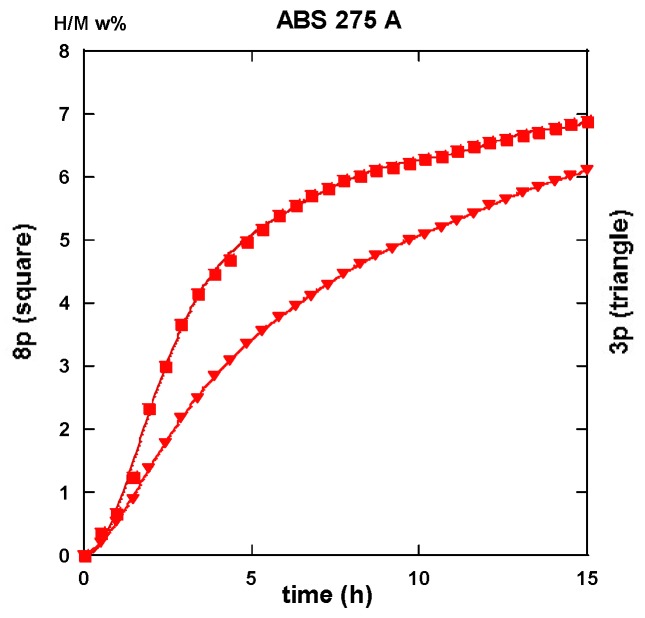
Hydrogen absorption rate (H/M w%) versus time at 275 °C (red) for 3 passes (triangles).

The traces shown in Figure 23a “3p.-175” and 23b “8p.-175” reveal marked differences in terms of absorption kinetics for which the three-passes show evidence of two incubation-steps. The first one occurs after less than half an hour while the other appears close to the 4 h reaction and a slightly accelerated slope is perceptible after the ~12 h reaction. The first incubation step remains visible after processing for eight passes. However, in both cases, only ~6 H w%, were absorbed in 15 h since the end part of the “8p.-175” trace indicates a more effective saturation. Quite similar trends are shown in Figure 24, which represents the absorption kinetics of the same materials after being treated at 275 °C for three and eight passes, respectively. A gain in kinetics is observed during the first 4 h of the incubation period, processing 3p. at 175 °C reference to 275 °C, but the uptake after 15 h is found to improve. Conversely, when processing for eight passes, H-absorption remains identical at both temperatures. However, it markedly increases after 4 h exposure for the 8-passes samples (15 h H-uptake ~7 H w%).

Both the gains in kinetics as well in hydrogen uptake for the eight passes processed a sample in comparison made with the three passes, take place after a rest time of almost ~2.5 h. This agrees well with the corresponding more uniform distribution of stresses, as determined by numerical simulation (Figure 9 and Figure 13).

**Figure 25:**
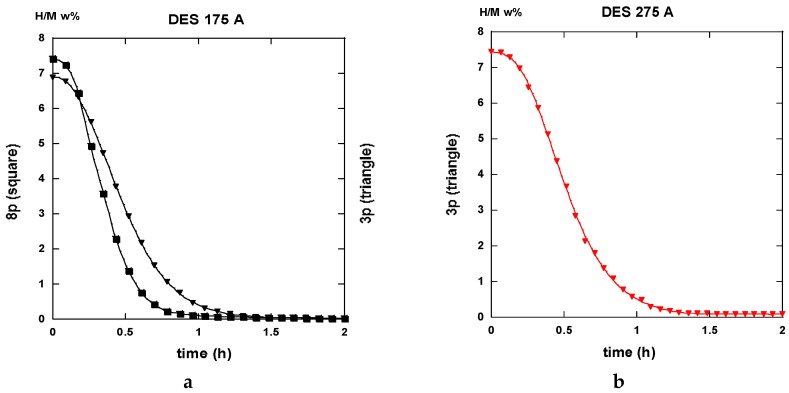
Hydrogen desorption rate (H/M w%) versus time of samples: (**a**)—for 3 passes (triangles) and for 8 passes (squares) as treated at 175 °C (black).; (**b**)—for 8 passes as treated at 275 °C (red).

After half an hour under desorption conditions, about 84% of hydrogen is released from the “8p.-175”. However, about 45% only from the “3p. 175” sample as shown in Figure 25a.

Interestingly, 50% of the hydrogen is released from the “3p.-275” sample as shown in Figure 25a,b. The “8p.-275” in the desorption process appears quite similar as it is for “8p.-175”.

It is worth it to retain that marked differences appear between the absorption traces recorded for three passes and eight passes. Mode A is the temperature of channeling −175 and 275 °C-in full agreement with the numerical simulation results e.g., Figure 3a,c show a delayed development and inhomogeneous density of strains in the sample at low pass numbers.

#### 2.3.2. Route B_C_

**Figure 26:**
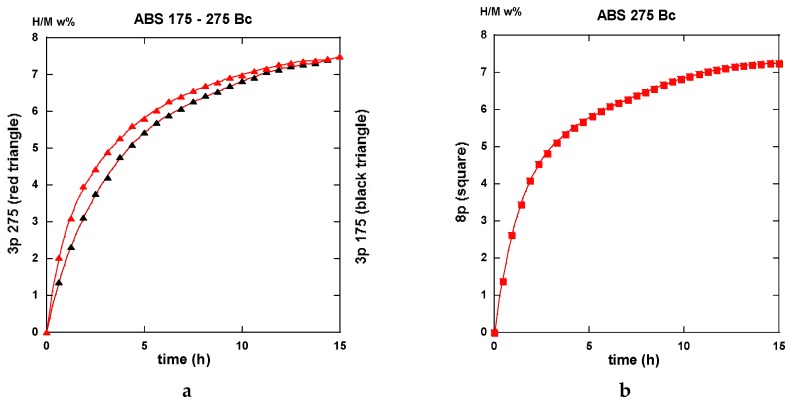
Hydrogen absorption rate (H/M w%) for B_C_ ECAP- treated billets: (**a**)—3 passes at 175 °C (black) and 3 passes at 275 °C (red). (**b**)—for 8 passes at 275 °C (red). Relative gains (kinetics and uptake) from three to 8 passes is less than for the A mode as shown in Figure 24 because of a more homogeneous micro-structure in the billet.

Conversely to all traces recorded for hydrogenation samples being processed via the A route which systematically exhibit an incubation phenomenon, all samples processed via the B_C_ route as well as an instantaneous and continuous hydrogen uptake is observed. For the three passes in processed samples, there is not so much difference in between the traces corresponding to 175 and 275 °C. As shown in Figure 26a in the intermediate period, the latter sample absorbs hydrogen somewhat faster, but, after 15 h, both samples appear almost saturated with ~7.5 H w%. Surprisingly the “8p.-175” sample reacts as faster as the “3p.-275” one up to 4 h exposure time, but it saturates less effectively in comparison with the two previous ones (~7 H w%), as shown in Figure 26b. Again, for the “8p.-275”, the kinetics is fairly similar since it was obtained for “8p.-175” up to 4,5 h of treatment, both saturating up to 7.2 H w% after 15 h.

**Figure 27:**
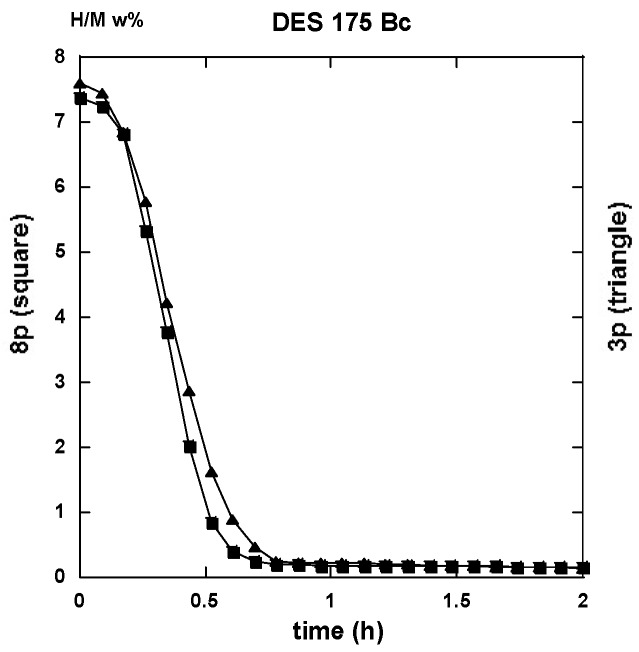
Hydrogen desorption rate (H/M w%) of samples ECAP-treated at 175 °C—2 passes (triangles) and 8 passes (squares).

**Figure 28:**
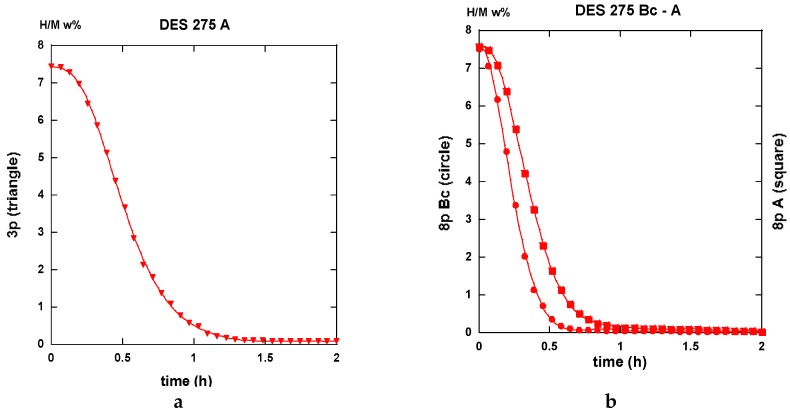
Hydrogen desorption rate (H/M w%) for: (**a**)—A-mode ECAP treated at 275 °C for 3 passes (**b**)—B_C_- and A-mode ECAP-treated for 8 passes at 275 °C.

Desorption operates rather similarly for both the samples treated at 175 °C for three passes and eight passes ECAP processes, respectively. This leads to 80% and 87% evacuated hydrogen after half an hour, as seen in Figure 27. If similar to what was obtained with the “8p.-175”, it is found better than the “3p.-175” processed via route A, as shown in Figure 25a. Figure 28a shows that the “3p.-275” is one of the less reactive at desorption since, after half an hour, only 40% hydrogen was desorbed. Conversely, both the “3p.-275” samples treated along the A and better the B_C_ routes are of the most desorbing ones with 80% and 95% (of the total hydrogen content), respectively, losses after half an hour, as shown in Figure 28b.

Conversely to the results gained with application of ECAP mode A, the samples processed using the mode BC exhibit less of a difference in the hydrogen absorption traces when passing the samples three and eight times, with less difference for whatever was the applied temperature. This agrees well with the numerical simulation approach. According to Figure 3b,d, mode B_C_ develops a strain density more effective and more homogeneous after early passes. The differences in terms of the resulting texture between mode A and B_C_ are well illustrated in Figure 18. It is obvious that both the strain rate and the nature of the texture could play determining roles on the hydrogenation process.

#### 2.3.3. Second Hydrogenation Cycles

**Figure 29:**
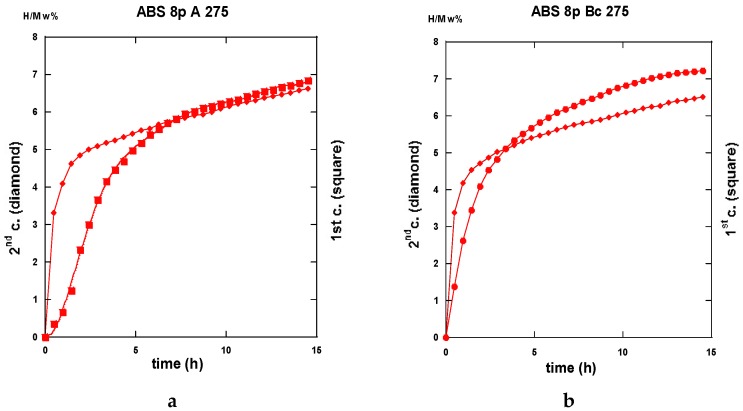
Comparison of the traces in terms of the 1st and 2nd absorption rates (H/M w%) of 8 passes ECAP-treated samples at 275 °C: (**a**)—mode A, (**b**)—mode B_C._

**Figure 30:**
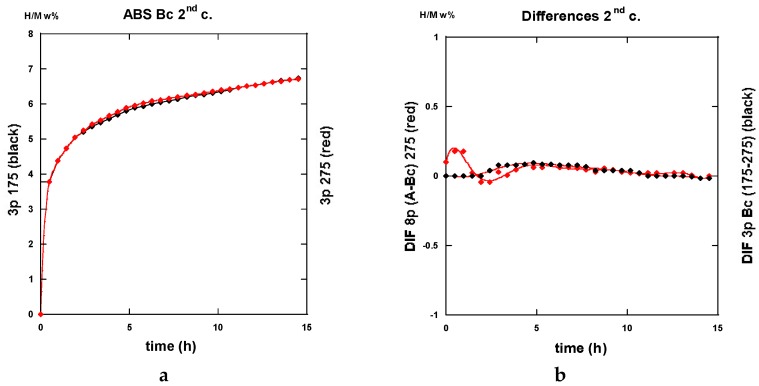
Second absorption rate traces (H/M w%) of: (**a**)—3 passes ECAP treated in B_C_ route at 175° and 275 °C: (**b**)—difference between traces A-B_C_ routes for 8 passes at 275 °C and for 3 passes B_C_ between 175 & 275 °C for similar results whatever the treatments are.

**Figure 31:**
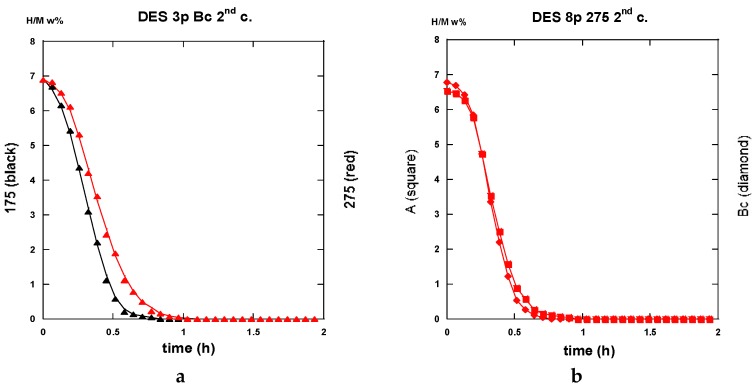
Comparison of the 2nd desorption rate traces (H/M w%) of: (**a**)—three passes B_C_ route ECAP treated at 175 and 275 °C. (**b**)—Eight passes ECAP-treated at 275 °C for A and B_C_ routes.

A few of the kinetics traces recorded after desorption and then for a second absorption process are given here after in the successive Figure 29, Figure 30 and Figure 31 allowing a direct comparison with thyose of forst absorption process. 

#### 2.3.4. Comparisons of Hydrogenation Processes

Perfect analyses for a comparison of the different sorption data by using various models of reaction kinetics, e.g., Jander, Johnson-Avrami-Melh-Kolmogorov (JAMK), Gistling-Broushtein or other expressions, were not made easy in spite of the apparently well shaped traces. There is one key assumption for deriving the corresponding equation for phase transformation kinetics is that the nuclei are distributed randomly in space. As shown in the numerical simulation part, the deformation density accumulated after a few numbers of ECAP passes is not made perfectly homogeneous within and along the sample, even if better after applying the B_C_ mode of channeling. However, from all the received data and the shown traces, it is worth it to note that:

1. All first hydrogenation processes reveal rather low kinetics of whatever is the applied ECAP route (A or B_C_), the number of passes (3 or 8), and the temperature of ECAP. This results mainly from the saturation approach for which the number of active nucleation centers diminishes. In fact, for the best cases, the t_50%_ (time for 50% uptake, theoretically H_max_ = 7.6 w% for pure Mg) is for 2 h or less.

2. Whatever are the ECAP conditions, all the 1st hydrogenation traces from mode A ECAP-treated samples reveal a S-curve kinetics i.e., an incubation phenomenon, that is found not affecting the B_C_ ECAP-treated samples, whatever were the applied conditions.

3. None of the 2nd absorption traces mode A reveal initially delayed values by such an incubation phenomenon. Thus, 50% of max uptake can be obtained in less than 1 h (e.g., three passes B_C_ at 175 °C or 275 °C, eight passes A and B_C_ at 275 °C). However, for the 2nd absorption cycle, the saturation approach appears less effective than for the first absorption one probably due to changes in the “annealed” microstructure (grain growth and less nucleation centers). This later phenomenon was well observed for other types of mechanical treatments (Ball Milling, High Pressure Torsion), which affects the following sorption processes. The max hydrogen uptake was restored after several tens of cycles [54].

4. For the same number of passes and the same temperature of ECAP-process, the route B_C_ reveals faster desorption than route A.

5. Of the best 1st desorption kinetics are realized with three and eight passes (little better) at 175 °C and eight passes at 275 °C (97% desorbed in 30 min), the situation is as good at the 2nd cycle for the three passes at 175 °C sample (95% desorbed in 30 min).

#### 2.3.5. Interpretation of the Hydrogenation Kinetics

Therefore, all the sorption traces were systematically analyzed in terms of Jander, GB, JAM laws [54]. The absorption kinetics relates the relative degree of achievement *ξ* to the time *t* with *k* being a coefficient model dependent.

nucleation-growth: (−ln(1 − *ξ*))^2^*= kt* (1)

3-D interface propagation at a constant diffusion surface area: (1 − (1 − *ξ*)^1/3^)^2^*= kt* (2)

interface propagation at a non-constant diffusion surface area: 1 − 2/3 *ξ* − (1 − *ξ*)^2/3^*= kt* (3)

contraction volume of the d-dimension surface: 1 − (1 *− ξ*)^1/*d*^ = *kt* (4)

**Figure 32:**
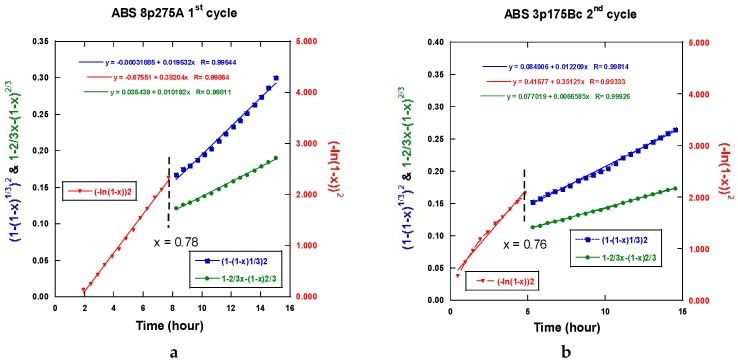
Numerical fits using Equation (1) at the beginning absorption for (2) and (3) at the end of absorption: (**a**)—for eight passes A mode treated at 275°C, the 1st cycle absorption, (**b**)—for three passes B_C_ mode treated at 175 °C, the 2nd cycle absorption.

No unique regime can be fitted from all the experimental absorption data. Figure 32a,b display two examples of fits realized accounting for Equation (1) at the beginning of the absorption reaction and Equations (2) or (3) for the end of the absorption reaction. First, a JMAK process takes place up to a limit value *ξ_L_*, then an interface propagation-diffusion mode is found. Both Jander and GB models look acceptable. 

A slightly better agreement is seen with the GB model for three passes ECAP and, conversely, the Jander model is slightly better for eight passes of the ECAP process. The main results with models (1) to (2) or (3) are reported in Table 1.

For both the A and B_C_ treated samples, the *k*-values respectively to Equations (1), (2), and (3) are found to increase with the number of passes and the ECAP-temperature at the first hydrogen absorption (except for the Jander mode 8p at B_C_. For the second hydrogen absorption, the *k*-JMAK value progress as well, but it remains quasi-constant for both the Jander and GB modes. It is worth noting that the *k*-values for the Jander mode are about 1.5 to 2 times larger than the corresponding *k*-values for the GB mode.

**Figure 33:**
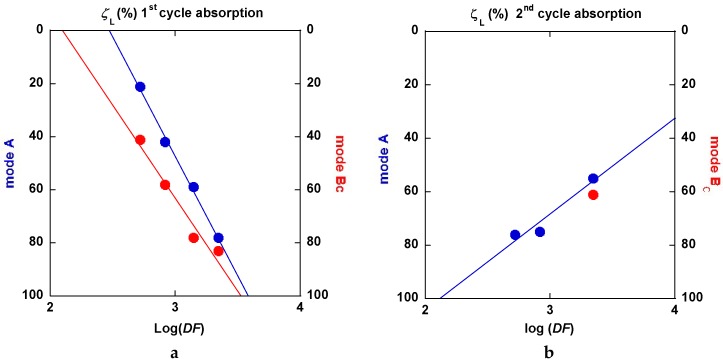
Limit value *ξ_L_* (%) of the change in kinetic process vs. log(*DF*). Blue dots are for ECAP mode A, red dots are for ECAP mode B_C_. (**a**): for the 1st hydrogenation (**b**): for the 2nd hydrogenation.

Reference to the classification of the samples (Table 1, column 1), the limit values *ξ_L_* at the first hydrogen absorption, both increase the parallel for passes mode A as well as for mode B_C_. However, it decreases at the second hydrogen absorption. The progressively continuous change of the value *ξ_L_* between a nucleation growth picture and an interface progression can be interpreted using the following information. Considering an effective “deformation” factor *DF* = np × T (number of passes × temperature of passes), it comes that the variations of *ξ_L_* versus log(*DF*) are linearly increasing for both mode A and B_C_ at the first hydrogenation cycle as shown in Figure 33a. The second one is more effective. At the second hydrogenation process, the variation of *ξ_L_* versus log(*DF*) linearly decreases as seen in Figure 33b. This means that the factor *DF* increases (exponentially) and the nucleation center when refining more and more the microstructure. 

Conversely, the first hydrogenation process at 350 °C acts as an annealing and grain growth process (probably from the finest ones grains and crystallites), which means the interface progression mode is again the dominant one. In fact, there is no s-type kinetic behavior at first and the approaches to saturation are referenced to those observed at the first hydrogenation.For the desorption regimes, once more it was not made possible to apply a unique desorption regime using the models of Equations (1) to (4). Since the desorption kinetics are not found to be much different under 10 kPa at 350 °C, for comparison, Table 2 gathers simple “characteristics” directly measured on the desorption traces. These characteristics have been extracted from both the first and second desorption processes.

As said above and shown in Figure 25a,b, Figure 27, Figure 28a,b, and Figure 31a,b, the desorption processes are rather fast since, in most cases, 85% to 90% of the hydrogen content is evacuated even if Table 2 reveals effective differences. A simple statistical analysis compares mean values and shows that, for the first desorption cycle for both: 1—mode A and B_C_ and ECAP temperature of 175 °C and 275 °C, the 8p mode reveals better than the 3p one for all four parameters, 2—numbers of passes and ECAP temperatures, the A and B_C_ modes reveals practically equivalent, 3—numbers of passes and the A and B_C_ modes, a 275 °C ECAP treatment reveals better than at 175 °C in terms of desorbed hydrogen uptake. Moreover, this results only from the previous hydrogen uptake during the absorption. In fact, as a result from a second absorption/desorption (H/D) cycle, the performances are as good as those exemplified here (either 8 passes, or 275 °C ECAP treatment), except for the maximum hydrogen uptake H_max_, which appears ~10 % weaker than at first. However, we anticipate that, after a limited but sufficient number of H/D cycles, the H_max_ will retrieve its full level close to 100% such as for other techniques of mechanical processing Mg and MgH_2_, as it was found and referenced in [55].

## 3. Discussion

3D (LS-Dyna) numerical simulations were realized for ECAP processing of the AZ31 magnesium alloy. It consisted in 1, 2, and up to 9 passes of ECAP with different orientations of the sample in between the successive pressings. This includes 1—the real stress-strain diagram, 2—the accumulation of plastic strain, and 3—the self-equilibrated residual stress field between passes. Fairly good correlations were established between the results of numerical modeling and the experimental deformation behavior of the materials. Computational methods have revealed interest to anticipate complex deformation behaviors of metal type samples submitted to ECAP deformation, which are an a priori guide to select the optimal SPD conditions.

It was shown that increasing the number of ECAP passes—mode A—results in the accumulation of residual strains (with gradual attenuation), which were non-uniformly distributed in the AZ31 sample. Options to rotate the sample in between ECAP passes—mode B_C_—leading to maximize the deformation (volume distribution function) were tested in agreement with known experiments. The level of accumulated residual stresses and their volume distribution functions were evaluated to be more effective. Up to three ECAP passes were applied at a temperature close. However, below the recrystallization temperature of AZ31, a minimum grain size (2–3 µm) microstructure was formed to be accompanied by a marked increase of micro-hardness. Both parameters remain almost constant after further ECAP cycles. Originally, the AZ31 alloy exhibits a strong strain hardening. It was transformed into an alloy with both a visible yield line and a smaller strain hardening. Although the intensity of strains continues to increase during further ECAP cycles, the residual stress density tends to saturate. In fact, after receiving 3 ECAP B_C_ passes, the transformation was considered advanced enough in forming a fine and rather homogeneous microstructure at minimized energy costs. This offers an interesting opportunity to achieve fast hydrogenation kinetics and form rapidly MgH_2_ [10,11,12,13].

Then, the microstructure transformation was investigated versus temperature and number of passes, focusing first on the ECAP B_C_ mode, which, from numerical simulations supported by micro-hardness analysis, had appeared to develop more rapidly as a higher and homogeneous density of stresses. A marked difference in the microstructure behavior was noticed to take place between 200 and 250 °C after 2-B_C_ passes only, which corresponds to the fragile/ductile behavior of the AZ31 type alloy. Figure 14 reveals that, at 150 °C for both ZK60 and AZ31 alloys and for 1 (A) to 2 (B_C_) passes respectively, the fracking regime had led to intense twinning mechanisms affecting the larger grain (A) and with a crossed field in thinner grains (mode B_C_). Then the very fine microstructure created at temperature above 200 °C had led to a homogeneous distribution of larger grains resulting in a recrystallization process. Effectively, after an ultima grain size refinement, the microstructure was homogeneously recrystallized in size using the mode B_C_. The passes number varies from 4 to 9. Therefore, the temperature and number of passes are two driving parameters enabling design of the grain boundary distribution. 

Williamson-Hall diagrams [44] were plotted from XRD patterns recorded perpendicular to the ECAP channeling to quantify both the mean size of crystallites (coherent volumes) and overall defects related to the strained matter. At room temperature and for 1 pass, the slope of the trace shown in Figure 15a is marked (large stress effect ~0.35%) and the mean size crystallite can be estimated of ~200 nm. When processing for 2 A-passes at 250 °C, the stress development was almost relaxed (no slope) as shown in Figure 15b and the crystallite size was decreased to <100 nm, which moreover exhibits an anisotropic shape distribution. The change of the strain rate bears out a parallel change of morphology observed on the grain shown Figure 12a,b for 2 B_C_ passes in the fragile and ductile states, respectively. Then, comparing the effect of a larger number of passes (9) at 250 °C as shown in Figure 15a and 16b, respectively, for the A and B_C_ modes, it was observed that the first one mode delivers a crystallite size of ~125 nm but of 300 nm for the second mode. Effectively, the mode B_C_ appears fully isotropic with a uniform and weak level of stress (0.025%). A similar situation was found for the (00l) direction and those rather parallel (0.025%), but the level of stress was found to be much larger at ~0.1% for the direction perpendicular to the c-axis. For both mode A and B_C_, a 10th pass was applied at room temperature and the mean respective crystallite size was calculated to be very close (125 and 100 nm, respectively, with a marked increase of the level of stress (0.3% and 0.2%, respectively). Mode BC appears more anisotropic than mode A with less distribution of the resulting values in reference to the crystal directions.

Al these results are fairly coherent with what was obtained using the Small Angle Neutron Scattering (SANS) technique (Figure 20), if applied to 1 pass only A at RT, which demonstrates anisotropic crystallite dimensions (300 × 100 nm) with some 4 to 8 nm size irregularities being more likely the signature of boundaries. Unfortunately, it was not possible to analyze different samples using SANS because an unpredicted schedule of events. 

Back to XRD techniques in terms of texture analysis, it was shown in Figure 17 for one pass that the grain and crystallites are markedly oriented in the ECAP process with a dominant (002) plane orientation. The crystal mean axial direction was found at the ~40° of the channel one. This deviation is clearly coherent with ECAP processing using a 105° angle of channeling. Moreover, when processing twice, mode BC is shown in Figure 18. The resulting maximum pole figure for (002) was found to be rotated by 45° in reference to the “A” mode (one pass), according to the 90° rotation of the sample of the B_C_ mode. The other main directions of the pole figure appear to be more extended and more diffuse in the pole figure. The sketch of texture integrated and normalized intensities of the four main crystal planes after one pass and after nine B_C_ passes (as shown in Figure 19) confirms and amplifies the previous information.

It was demonstrated that, when applied several times, the B_C_ mode leads deliver a better homogeneous crystallites size (~200–300 nm) and their orientation within the billet. In terms of homogeneity and size distribution of the grains, it appears more effective to process at a temperature of 250 °C, which is above the fragile/ductile transformation temperature of AZ31.

The texturation process of a hexagonal-type structure is not as simple as for cubic ones. It was studied in more details [42,55,56] and after applying SPD techniques such as ECAP [27]. It is conditioned by the ability of the material to accumulate stresses by deformation mechanisms with the developments of dislocations, slip bands, and twinning. In addition, this directly depends on the thermal variation of the Young modulus and parameters related to the elastic energy. If the anomalies detected and shown in Figure 21 using the anelastic spectroscopy on both the Young modulus and elastic energy dissipation were not definitively identified and explained, one should suggest that the dislocation motion created during RT temperature processing can move effectively close to 360 K in the range of a few Hz excitation. However, deeper experimental analysis must be undertaken.

Inelastic spectroscopy is one of the best tools to quantify the thermal induced diffusion of elements inserted in solids (e.g., H, C, N…). This type of technique was applied on MgH_2_ based materials, but this can be applied on a consolidated powder sample since hydrogenation leads to a well-known decrepitation process. Plots of the elastic energy dissipation factor (Q^−1^) with excitation in the kHz range (according to the intrinsic diffusion rate of hydrogen in Mg) do not reveal any peak singularity and the relative variation of the dynamic Young modulus (ΔE/E) present a monotonic increase when the temperature decreases. Then it was observed that Q^−1^ values are almost temperature independent until 650 K because the relaxation rate of hydrogen is too low to be detected by AS. 

Then a systematic analysis of the kinetics of hydrogenation/dehydrogenation was realized on all the different AZ31 ECAP treated samples at 175 and 275 °C after three and eight passes in mode A and B_C_, respectively. These studies reveal that, for the first hydrogenation, all the A ECAP-treated material exhibit a clear incubation process for at least one hour at 350 °C under a 2 MPa H_2_ pressure. Conversely, none of the B_C_ ECAP-treated samples undergo such a delaying condition, especially with a better intermediate kinetic rate and a slightly better approach to saturation. Such a difference should be addressed to the difference in homogeneity and distribution of stresses and particle size refinement, the texturation effects that leads to a more anisotropic crystallite shape and stress distribution for A-processed sample than for B_C_ ones.

However, after desorption and for a second hydrogenation cycle, the different samples exhibit more similar kinetic rates of absorption without any incubation time (A samples). In all cases, none of the typical models (JMAK, constant diffusion, interface propagation) were found to describe the whole kinetic profile experimentally established. This is probably due to remaining dispersion in terms of stress and microstructure development whatever ECAP conditions were applied. For almost all analyzed results, a nucleation-propagation growth type (JMAK) takes place by following a degree of completion *ξ_L_*, by a Jander-type model of diffusion-interface-propagation phenomenon. A similar mechanism has been shown to take place for the second hydrogenation process. Defining a driving factor *DF* for the limit index *ξ_L_*, as *DF* = (nb ECAP passes × temperature of process), a linear variation was fairly established between log (*DF*) and *ξ_L_*. This allows for the relation of the crystallites micro-structuration reference to the operating mechanism for hydrogenation. As shown in Figure 33a for the 1st hydrogenation cycle, *ξ_L_* decreases linearly and fast with log(*DF*). This means that more and more hydrogenation proceeds via a diffusion-propagation interface via a better refinement of the microstructure with the ECAP pass number and temperature. Mode B_C_ looks more effective than mode A, but, when the factor *DF* increases (eight passes-275 °C), the difference is weaker and weaker. For the second hydrogenation cycle, the limit index *ξ_L_* versus log(*DF*) criteria exhibits a positive slope, which means that the incubation-propagation mode prevails more and more effectively, as shown in Figure 33b. In fact, all second absorption traces reveal to be more effective at the beginning while being less effective when approaching the H_max_ and when compared to what was found at the first cycle. The initial nucleation-propagation mode was made more efficient (memory effect vs nucleation centers) during the first hydrogenation/dehydrogenation cycle, which is a limited recrystallization phenomenon that was induced by annealing at 350 °C. This somewhat modifies the final hydrogenation kinetics. Such a tendency breaking the absorption capacities was already found for different mechanical processes of MgH_2_, but, after a few cycles, a full hydrogen absorption capacity was retrieved [57].

At desorption levels, the differences between all the studied traces were found to be less marked since almost of the samples were 90% desorbed in less than 30 min. For all cases, the kinetic of desorption was found to be improved during the second cycle. The best performances were achieved with maximum values of the driving factor *DF* (max ECAP passes, highest temperature).

ECAP processing of the AZ31 Mg alloy was demonstrated suitable for application in view of MgH_2_ mass production. An optimized and reactive micro-structure and nano-structure can be received using selected routes and parameter processes. However, better achievements in terms of kinetics can be expected by processing specifically formulated Mg-alloys and specially designed dies to optimize the mass production at low costs (time, man-power, energy).

## 4. Materials and Methods

The materials used for the present investigation were a commercial AZ31 alloy from Magnesium Elektron (Clifton, Swinton, Manchester, UK), ZK60 kindly provided by V. Skripnyuk (Technion, Haifa, Israel), and pure Mg and MgH_2_ powder from McPhy Energy SA (La Motte Fanjas, France). 

**Figure 34:**
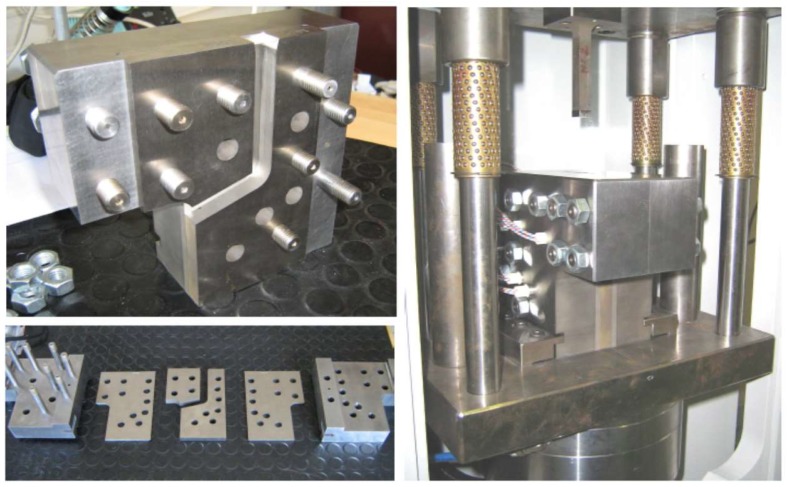
ECAP tool used for SPD treatments to Mg alloys, as developed by POINSARD Design and Tool SAS (Besançon, France). Up and left: the open channeling die, down-left: tools forming different angle channels, Φ_E_ = 95°, 105°, 115°, 125°, and 135°, right: ECAP system (die, punch) [36].

Samples submitted to ECAP process were 10 × 10 × 50 mm parallelepiped billets cut from bars by spark erosion. The used ECAP tool is pictured in Figure 34. Specific sensors allow record versus time as well as both the effort of the press and the relative displacement of the punch to the die during the ECAP process. In addition, six heaters and one thermocouple inserted in the die walls are connected to a temperature controller.

In order to visualize the final distribution of deformation strains at the surface of the billet, prior to the ECAP treatments, a square array was drawn on the four 10 × 50 faces of the billets and shown to be deformed in Figure 1 after one ECAP process. Severe Plastic Deformation of the billets was carried out via the A and B_C_ routes in the ECAP die with a channel-crossing angle of Φ_E_ = 105°. This angle was selected as of the most SPD effective (close to 90°) and was found to deliver less surface cracked samples. In fact, to avoid or to minimize the friction forces with the walls of the channel, a carbon lubricant spray MOTIP^®^ (MOTIP DUPLI B.V, Wolvega, The Netherlands) was applied to the billet before channeling. Pieces of the samples used for the microstructure analyses were square plates of 10 × 10 × 2 mm cut from the top, the middle, and the end parts of the AZ31 billet, which was perpendicular to the axis of channeling. The microstructure analyses were performed using both optical and scanning electron microscopes of type METAM LP-32 (HILTI, Schaan, Germany) and Hitachi S3400 (HITACHI High Technology, Tokyo, Japan), respectively. The grain size was calculated by the method of cross-sections based on optical microscopy data.

Microhardness was measured at room temperature by the Vickers method using a digital automatic hardness tester DM-8 Affri (OMAG di Affri D.&C. sas, Varese, Italy) under a 1 N load with a load time of 10 s. Uniaxial compression tests of parts of the billet samples (~10 × 10 × 10 mm) were operated at room temperature using a universal testing machine Zwick/Roell Z-250 (Zwick/Roell, Ulm-Einsingen, Germany) in a constant crosshead speed mode of about 1 mm/minute. The direction of the mechanical load applied to the samples coincided with the axis of channeling during the ECAP process. 

3D numerical simulations of the ECAP processed billets were carried out using the LS-Dyna package (Livermore Software Technology Corporation, Livermore, CA, USA, www.lstc.com) developed under the ANSYS^®^ software package (ANSYS, Canonsburg, PE, USA) that allows implementations of the explicit and implicit variants of Finite Element Modeling (FEM) using Lagrangian and Eulerian meshes, respectively. The parallelepiped billet in direct and hard contact of the punch was assumed to be moving within the rigid channels of the die (channeling angle at 105°) without friction to the walls at a predetermined initial speed of about 5 cm/s. An elastic-plastic model with a yield surface function for Mises isotropic hardening [37,38] was used (Piecewise Linear Plasticity). The hardening parameters were taken from experiments realized on the AZ31 alloy at room temperature [35,37,38].

X-ray diffraction patterns were recorded using Siemens D5000R equipment (SIEMENS/BRUCKER, Karlsruhe, Germany) with a θ-θ geometry diffractometer working at λ_CoKα_ radiation in reflection mode. For powder samples, a PW-3830-Philips equipment (Malvern Panalytical, Eindhoven, The Netherlands) was used with a transmission diffractometer in Brent-Brentano mode, working at λ_CuKα_ radiation and equipped with a graphite monochromator. The sample-holders can be used in a rotating mode to minimize any preferential orientation effects.

Pole figures were established on bulk samples using an X-ray Siefert goniometer (GE Sensing & Inspection Technologies GmbH, Arhensburg, Germany) equipped with a W/Si multilayer collimator (2D Xenocs) and Si analyzer. The experimental data were recorded at λ_CuKα_ radiation and then treated with the LaboTex program for texture analyses and to evaluate the orientation distribution function (ODF) as well as for the generation of inverse pole figures.

Small Angle Neutron Scattering (SANS) experiments were undertaken at the Institute Laue Langevin–ILL, France), using the D11 instrument facilities.

Microstructural characterization was carried out by using an Olympus BX51 optical microscope (OLYMPUS, Tokyo, Japan) with polarized light for interference contrast. Transmission electron microcopy (TEM) analysis was carried out in Philips CM 300 microscope (Philips, Eindhoven, The Netherlands) on samples electrochemically polished using a TENUPOL 5.0 equipment (STRUERS SAS, Champigny sur Marne, France).

Anelastic Spectroscopy enabling recorded the effects of lattice distortion defects in terms of the coefficient of energy dissipation and elastic modulus, which both recorded the temperature. For that, the sample is mounted on a flexural-torsional-extensional vibration-meter (homemade equiments) and activated by an alternating stress electrode vibrating in Hz (metal) [50] or kHz (hydride) range [49].

Systematic kinetic hydrogen absorption/desorption as well as hydrogen up-take measurements were carried out using two Sieverts type apparatuses, a PCI HERA C2-3000 (HERA Hydrogen Storage, Longueil, QU, Canada) and a HyEnergy PCTPro-2000 (SETARAM, Caluire et Cuire, France), which applied H_2_ pressure at various temperatures.

## 5. Conclusions

In the present work, a triple stage analysis of the ECAP processing Mg and its alloys (e.g., AZ31) was undertaken in view to form efficiently MgH_2_ for mass hydrogen storage.

First, a numerical simulation method was applied to predict and compare the effectiveness of the two main modes of ECAP—A and B_C_ passes—in terms of grain size refinement and stress density. The number of successive passes and the temperature of the samples were the two determining parameters. Microhardness measurements were used as a border guide to assert the numerical derivations.

Second, a panel of techniques such as metallography, SEM, XRD, texture analysis, SANS, and Anelastic spectroscopy investigations were successively enforced, which allowed for establishing fair agreements between the calculated characteristics and their experimental determinations. Effectively, the grain size and the crystallite size distribution, the stress density and orientation of the main crystal axis were all parameters of interest to better understand further interaction with hydrogen.

Third, the hydrogenation/dehydrogenation kinetics and the hydrogen uptake were systematically quantified in reference to the various ECAP process (mode A or B_C_, number of passes, temperature of ECAP process, 1st to 2nd HD cycle). Depending on the number and the temperature of passes, it is derived that a nucleation-propagation mechanism leads to a wall-propagation mechanism, describing the Mg to MgH_2_ transformation. Definitively, it is shown that the best hydrogen sorption performances are rapidly better derived from the B_C_ mode than the A one. However, it is demonstrated that an appropriate number of passes at a temperature high enough delivers interestingly activated materials for practical hydrogen storage for large applications. In fact, for large storage units, the kinetic of reaction is not the most important parameter to consider since, during a real situation, this is the thermal management in tank of the exothermic/endothermic reactions that control the hydrogenation/dehydrogenation operations [22,23,24].

However, it can be suggested that definitively superior performance materials can be achieved by specific improvements: 1—If the starting alloys contain of the most efficient additives [58], 2—With work-piece instrument experiencing, successive В_С_ passes without losing cohesion, which can be found in ref. [41].

## Figures and Tables

**Table 1:** The *k* (H^−1^) parameters and limit values ξ_L_ (%) of the kinetic process after fitting the various traces recorded for the 1st and 2nd hydrogen absorption and for the different ECAP-treated samples.

Samples	1st Absorption				2nd Absorption			
	JMAK	ξ_L_	Jander	GB	JMAK	ξ_L_	Jander	GB
3pA175	-	0.21	0.011	0.008	X	X	X	X
3pBc175	0.192	0.41	0.033	0.019	-	0.76	0.012	0.007
3pA275	0.098	0.42	0.014	0.009	X	X	X	X
3pBc275	0.361	0.58	0.037	0.019	0.409	0.75	0.011	0.006
8pA175	0.287	0.59	-	-	X	X	X	X
8pBc175	0.348	0.78	0.022	0.011	X	X	X	X
8pA275	0.382	0.78	0.019	0.010	0.600	0.61	0.012	0.007
8pBc275	0.468	0.83	0.033	0.015	0.658	0.55	0.012	0.007

**Table 2:** Graphical parameters collected from desorption traces—Slope of the linear part. Initial Time (~incubation). End Time from linear extrapolation to H = 0. Desorbed amount relative to H_max_. X means that no sample was achieved for.

Samples	1st Desorption				2nd Desorption			
	Angle of Slope (Degrees)	Initial Time (min)	End Time (min)	Desorbed H/H_max_ (%)	Angle of Slope (Degrees)	Initial Time (min)	End Time (min)	Desorbed H/H_max_ (%)
3pA175	75	9.5	49	0.92	X	X	X	X
3pBc175	76.5	10.5	31	0.85	78	11	40	0.90
3pA275	76	19	48	0.98	X	X	X	X
3pBc275	74.5	16.5	53	0.98	79	9	32	0.88
8pA175	79	6	34	0.98	X	X	X	X
8pBc175	80	9.5	33.5	0.94	X	X	X	X
8pA275	77.5	9.5	21	0.98	81	7	29	0.90
8pBc275	81	5.5	18	0.99	82	8	29	0.92
